# High Glucose Promotes Aβ Production by Inhibiting APP Degradation

**DOI:** 10.1371/journal.pone.0069824

**Published:** 2013-07-23

**Authors:** Yi Yang, Yili Wu, Shuting Zhang, Weihong Song

**Affiliations:** 1 Townsend Family Laboratories, Department of Psychiatry, Brain Research Center, Graduate Program in Neuroscience, The University of British Columbia, Vancouver, Canada; 2 The Ministry of Education Key Laboratory of Child Development and Disorders, and Chongqing City Key Laboratory of Translational Medical Research in Cognitive Development and Learning and Memory Disorders, Children’s Hospital of Chongqing Medical University, Chongqing, China; Cleveland Clnic Foundation, United States of America

## Abstract

Abnormal deposition of neuriticplaques is the uniqueneuropathological hallmark of Alzheimer’s disease (AD).Amyloid β protein (Aβ), the major component of plaques, is generated from sequential cleavage of amyloidβ precursor protein (APP) by β-secretase and γ-secretase complex. Patients with diabetes mellitus (DM), characterized by chronic hyperglycemia,have increased risk of AD development.However, the role of high blood glucose in APP processing and Aβ generation remains elusive. In this study, we investigated the effect of high glucose on APP metabolism and Aβ generation in cultured human cells. We found that high glucose treatment significantly increased APP protein level in both neuronal-like and non-neuronal cells, and promoted Aβ generation. Furthermore, we found that high glucose-induced increase of APP level was not due to enhancement of *APP* gene transcription but resulted from inhibition of APP protein degradation. Taken together, our data indicated that hyperglycemia could promote AD pathogenesis by inhibiting APP degradation and enhancing Aβ production. More importantly, the elevation of APP level and Aβ generation by high glucose was caused by reduction of APP turnover rate.Thus,our study provides a molecular mechanism of increased risk of developing AD in patients withDMand suggests thatglycemic control might be potentially beneficial for reducing the incidence of AD in diabetic patients and delaying the AD progression.

## Introduction

Alzheimer’s disease (AD) is the most common neurodegenerative disorder characterized by a progressive decline in memory and other cognitive functions.It is estimated that over 35 million people worldwide suffered from the disease and AD-related healthcare cost was$604 billion in 2010 alone [1], [2].While early-onset AD can be caused by gene mutation in *APP*, *presenilin1* or *presenilin2,* it only accounts for less than 5% of all AD cases [3]. The majority of AD cases are sporadic usually with late-onset, caused by unelucidatedmechanisms. Both early-onset AD and late-onset AD are characterized by the common major neuropathological features including neuriticplaques, neurofibrillary tangles, and neuronal loss. Amyloid β protein (Aβ), the major component of plaques, derives from sequential cleavage of amyloidβ precursor protein (APP) by β-secretase and γ-secretase complex.

APP is a type I integral membrane protein, encoded by *APP* gene on chromosome 21 [4]–[7]. There are three major isoforms of APPderived from alternative splicing, of 695 (APP_695_), 751 (APP_751_) and 770 (APP_770_) amino acids, respectively. APP_695_ is predominant in the neurons of the brain, while APP_751_and APP_770_are more ubiquitously expressed in most tissues such as kidney, lung, and muscle [8]–[10].APP expression is regulated at multiple levels including transcription, translation and post-translation [11]. *APP* gene transcription is governed by a complex promoter [12], [13] and subjected to the regulation of many transcription factors such as homeobox protein Hox-1.3 and NF-κB [14], [15]. APP expression can be stimulated by interleukin-1, retinoic acid, phorbol esters, growth factors [16]–[19] and various stresses including heat shock, treatment with ethanol and sodium arsenite [20], [21]. In addition, APP is extensively post-translationally modified including N-glycosylation, O-glycosylation, tyrosine sulfation and phosphorylation [22], [23] and is detected in various subcellular locations endoplasmic reticulum, Golgi apparatus, and plasma membrane [24]–[27]. APP is metabolized in lysosomes [28], [29],and recent study indicates it also being degradedthrough ubiquitin-protesome pathway [30].The half lifeof APP is 20–30 minutes [23]. It has been reported that APP degradation and processing is significantly affected by its glycosylation modification [31].

APP can be cleaved by β-site APP cleaving enzyme 1 (BACE1) at two sites of Aβ domain, Asp^1^ and Glu^11^, generating C-terminal fragments of 99 or 89 amino acids (C99 or C89) respectively [32], [33]. C99 is subsequently cleaved by γ-secretase complex in the transmembrane domain, liberating Aβ and APP intracellular domain. Thus, inhibition of BACE1 and/or γ-secretaseactivity might be an effective way for AD treatment by reducing Aβ production [34]–[36].Yetunder normal conditions, majority of APP protein are cleaved by α-secretase within the Aβ domain, generating a secreted N-terminal fragment (sAPP_α_) and a membrane-bound C-terminal fragment of 83 amino acids (C83) [37], [38], which excludes Aβ generation.

Althoughrecent studies reveal that APP is involved inbrain development and repair [39], [40], the most well-known function of APP is its pathologic role in AD development. *APP* gene mutation is the first identified genetic mutation that causes early-onset AD [41]. In addition, elevated APP expression is implicated in the pathogenesis of both early-onset and late-onset AD. First, Down syndrome (DS) patients caused by trisomy of chromosome 21 show higher APP expression in brains compared to control cases and they inevitably develop AD after middle age, although increased expression of other chromosome 21genes might also contribute to AD pathogenesis, such as *RCAN1* [42]–[45]. Second, APP overexpression either through gene duplication or mutations in *APP* promoter can also cause early-onset AD [46], [47]. Importantly, recent studies suggest that APP upregulationis also involved in late-onset AD. MiR-106b, which can downregulate APP expression, was found significantly decreased in sporadic AD [48] and marked increase of *APP* mRNA has been reported in sporadic AD [49].Taken together, these studies indicate that dysregulation of APP expression and processing plays a central role in AD pathogenesis.

Diabetes mellitus (DM) is a complex metabolic disorder featured by chronic hyperglycemia. Accumulating epidemiological evidence shows a 50–100% increase in risk of developing AD in patients with DM [50], [51]. Diabetic patients exhibit cognitive deficits including damaged verbal memory, diminished mental speed and mental flexibility [52], [53]. Moreover, recent studies have shown that chronic hyperglycemia, the cardinal feature of diabetes, could acceleratethe development of AD pathologyin AD model mice.In diabetic mice induced by streptozotocin injection, the formation of both neuritic plaques and neurofibrillary tangleswere potentiated [54], [55].On the other hand, it has been reported that 81% of the AD patients show eitherimpaired fasting glucoseor frank diabetes [56]. Although a large body of data suggests that AD is highly associated with DM, the underlying mechanisms of this association are still unknown.

In this study, we first examined the effect of high glucose on APP metabolism and Aβ production *in vitro*. We found that high glucose significantly increased APP level and Aβ generation. Furthermore, we found that high glucose-induced increasein APP level was not due to enhancement of its gene transcription but resulted from inhibition of its protein degradation. Our data indicated that increased APP level and Aβ production by high glucose is caused by reduction of APP turnover rate. Thus, our study not only provides a molecular mechanism underlying the increased risk of developing AD in DM patients but also suggests that glycemic control might be potentially beneficial for reducing the incidence of AD in people with DM and delaying the AD progression.

## Methods

### Cell Culture, High Glucose Treatment and CHX Treatment

Human neuroblastoma cells, SH-SY5Y (ATCC CRL-2266), were maintained in glucose-free Dulbecco’s modified eagle medium (DMEM) supplemented with 2.5 mM D-glucose, 10% fetal bovine serum (FBS), 50 units of Penicillin and 50 µg of Streptomycin (Life Technologies). 20E2 cells, a cell line of human embryonic kidney293 cells, HEK293 (ATCC CRL-1573), stably expressing Swedish mutant APP_695_ generated in our lab previously [57], were cultured in glucose-free DMEM supplemented with 5.5 mM D-glucose, 10% FBS, 50 units of Penicillin and 50 µg of Streptomycin and 50 µg/ml geneticin. All cells were maintained at 37°C in an incubator containing 5% CO2. High glucose media were prepared by adding additional glucose to 10 and 25 mM glucose. The osmotic pressure was adjusted with D-mannitol (Fisher). The cells were treated with high glucose media for 24 or 48 hours. For APP degradation experiment(half-life measurement), 20E2 cells treated with media containing high glucose and cycloheximide (CHX) at 100 ug/ml (Sigma) were harvested at 0, 15, 30 or 60 minutes for western blot analysis. Similarly, SH-SY5Y cells were treated with high glucose media containingcycloheximide for 30minutes and then harvested for western blot analysis.

### Luciferase Assay

ThepAPP-Luc plasmid, containing 2.94 kb of human *APP* promoter region upstream of the firefly luciferase reporter gene [32], was used for luciferase assay to determine the activity of *APP* promoter. The transfection was performed using Lipofectamine 2000 (Invitrogen). One day prior to transfection, SH-SY5Y cells were seeded onto 60 mm plates at the density of 5.0×10^5^cells/ml culture media. On the day of transfection, the cells were grown to approximately 70% confluence and transfected with 4.6 ug pAPP-Luc plasmid DNA along with 18.4 ng*Renilla* luciferase vector pCMV-Rluc (Promega) to control transfection efficiency. Four hours after transfection, the cells were seeded to 24-well plates and grown overnight. 16 to 18 hours later, the culture media were changed to conditional media with different glucose concentration. The cells were treated for 24 or 48 hours and then harvested. The luciferase assay was performed according to the protocol for Dual-Luciferase Reporter Assay system (Promega) using a luminometer (Fluoroskan Ascent, ThermoLab Systems).

### Semi-quantitative Reverse Transcription PCR

RNA was extracted from cells using TRI-Reagent (Sigma). An equal amount of RNA samples were used as templates to synthesize the first strand cDNA with ThermoScript™ reverse transcriptase (Invitrogen). The newly synthesized cDNA then served as templates and the coding sequence of human APP and β-actin was amplified by Platinum Tag DNA polymerase (Invitrogen) with following primers: APP forward 5′-gctggcctgctggctgaacc;APP reverse 5′-ggcgacggtgtgccagtgaa;β-actin forward 5′ –cgaggatccggacttcgagcaagagatgg;β-actin reverse 5′-cagtctagagaagcatttgcggtggacg.β-actin levels served as an internal control. The PCR products were analyzed in 0.8% agarose gel (Sigma).

### Western Blotting

Cells were harvested and lysed in RIPA-DOC lysis buffer (0.05 M Tris-HCl pH 7.2, 0.15 M NaCl, 0.1% SDS, 1% sodium deoxycholate, and 1% triton x-100) supplemented with complete protease inhibitor (Roche Diagnostics). Lysates were sonicated and centrifuged at 13,200 rpm for 10 minutes to pellet the cellular debris.The supernatant was then diluted in 4X SDS-sample buffer and boiled. After resolved in 8% tris-glycine SDS-PAGE, the proteins were transferred to polyvindylidine fluoride (PVDF-FL) membranes (Immobilon-FL, Millipore, MA, USA). For immunoblot analysis, membranes were blocked with 5% non-fat milk dissolved in phosphate-buffered saline (PBS) for 1 h and incubated in primary antibodies overnight at 4°C. Antibody C20 is a polyclonal rabbit antibody made in-house that recognizes the lasttwenty amino acids of the APP carboxyl-terminus and was used to detect APP protein and C99 fragment. Monoclonal antibody AC-15 (Sigma) was used to detect β-actin. After incubation, the membranes were washed in PBS with 0.1% Tween-20 and incubated with secondary antibodies, IRDye™ 680 goat anti-rabbit antibody or IRDye™ 800CW-labelled goat anti-mouse antibody (LI-COR Biosciences) at room temperature for 1 hour, and visualized using an Odyssey Infrared Imaging System (LI-COR Biosciences).

### Aβ ELISA

24 hours after high glucose treatment, conditioned cell culture media of 20E2 cellswere collected. 20E2 cells are HEK293 cells that stably expresses Swedish mutant APP_695_.To prevent degradation of Aβ, protease inhibitors (AEBSF) were added. The cell media were centrifuged at 2000 rpm for 5 minutes to precipitate cells in the media. The concentration of Aβ40 was measured by Aβ40 human ELISA kit (KHB3482, Life Technologies) according to the manufacturer’s instructions.

### Statistics Analysis

Three or more independent experiments were performed. For APP degradation experiment, two-way ANOVA followed by Bonferroni test was used for statistical analysis. The rest of data were analyzed by one-way ANOVA followed byTukey test. Values were expressed as mean±S.E.M. We reported F value and P value, and P<0.05 was considered as statistically significant.

## Results

### High Glucose Treatment Increases APP Protein Level

**Figure 1:**
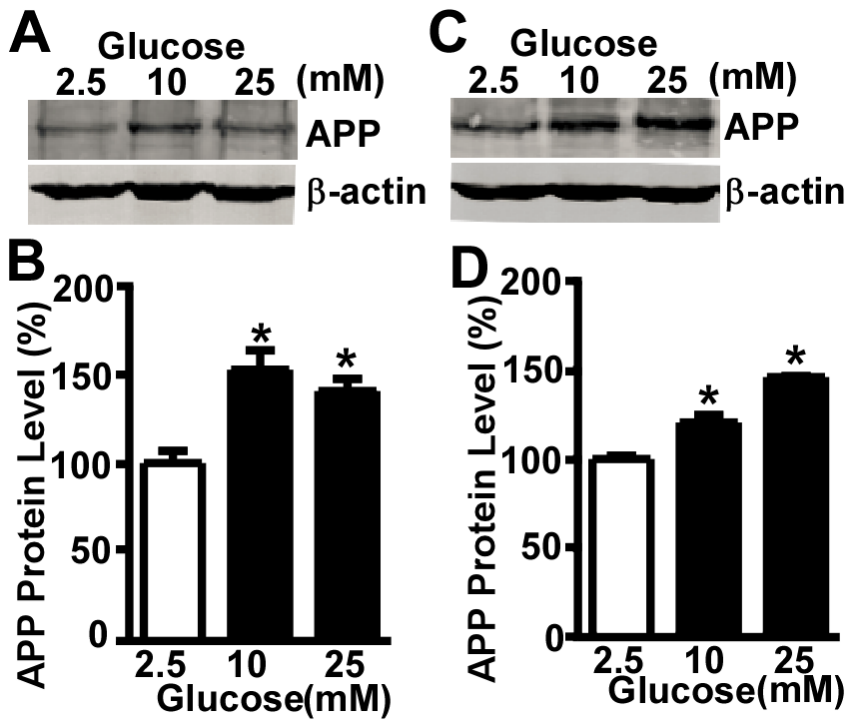
High glucose treatment increases full-length APP protein level. SH-SY5Y cells were treated with different concentration of glucose for 24 hours (**A**), and 48 hours(**C**) with2.5 mM glucose treatment serving as control. The cell lysates were analyzed by Western blot. Full-length APP was detected by C20 antibody. β-actin, serving as internal control, was detected by AC-15 antibody. 24-hour and 48-hour of high glucose treatment significantly increased full-length APP protein level.Quantification of full-length APP after 24-hour treatment of high glucose in SH-SY5Y cells (**B**). The values are expressed as mean±S.E.M, n = 4,*p<0.05 by ANOVA. Quantification of full-length APP after 48-hour treatment of high glucose in SH-SY5Y cells (**D**) The values are expressed as mean±S.E.M, n = 3,*p<0.05 by ANOVA.

Toinvestigate whether hyperglycemia could affect APP level, we treated the human neuroblastoma SH-SY5Y cells with media containing 10 mM or 25 mM glucose to mimic hyperglycemia*in vitro* and compared with cells treated with 2.5 mM glucose media which served as control. The 2.5 mM glucose was used as the equivalent of the physiological extracellular glucoseconcentration in the human brain [58], [59]. We found that 10 or 25 mM glucose treatment significantly increased the level of full-length APP protein to 152.63±10.78% or 140.59±6.80% (F = 10.88, p<0.05), respectively,comparing with the control at 24-hour time point (Fig. 1A and B). Similarly, the level of full-length APP was significantly increased to 120.52±4.20% or 146.04±0.59% (F = 74.5, p<0.05), respectively, in cells treated with 10 or 25 mM glucose for 48 hours comparing with the control (Fig. 1C and D).

### High Glucose Treatment does not affect *APP* Gene Transcription

**Figure 2:**
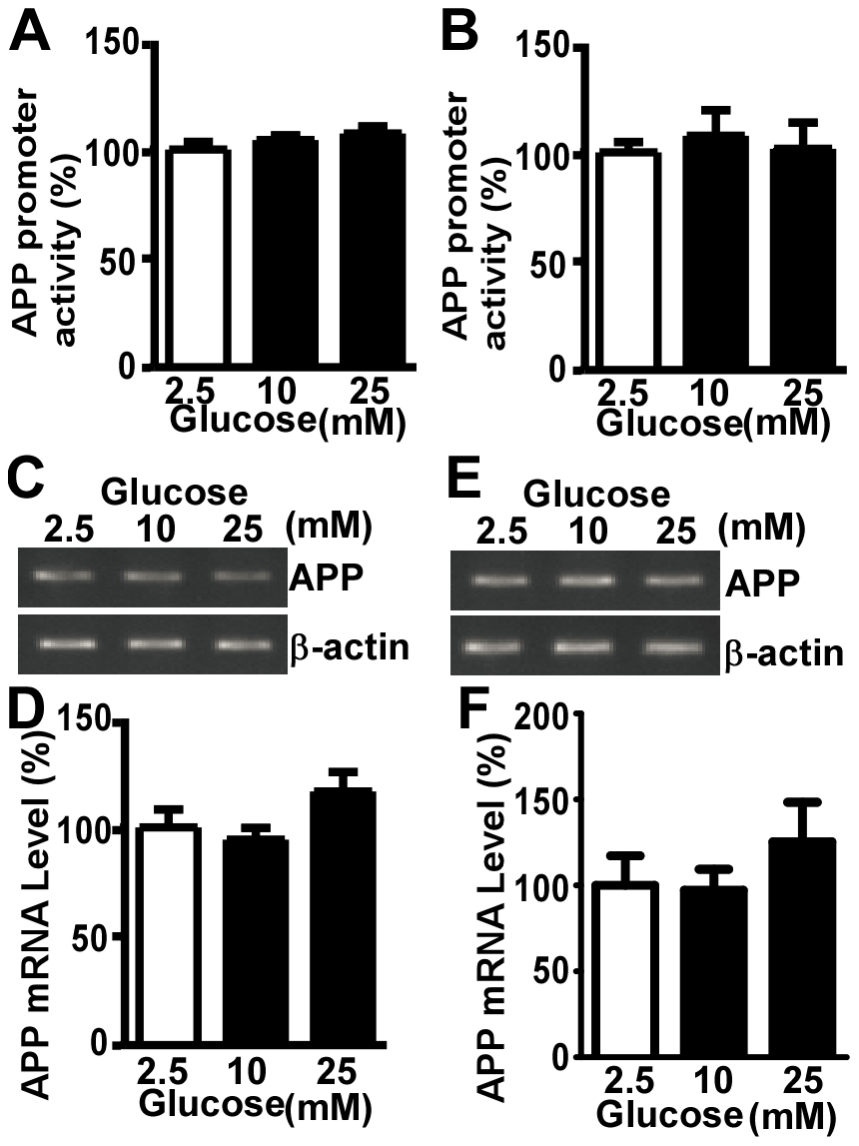
High glucose treatment does not affect APP transcription. The 2.94 kb human APP promoter was transfected into SH-SY5Ycells and treated with high glucose for 24 hours (**A**) and 48 hours (**B**). 2.5 mM glucose serves as control. Luciferase assasy was performed.High glucose treatment did not affect APP promoter activity. All the promoter data shown are results of 4 independent experiments, with each condition performed in triplicates. The values are expressed as mean±S.E.M. n = 4, by ANOVA. SH-SY5Y cells were treated with different concentration of glucose for 24 hours(**C**) and 48 hours (**E**). RNA was extracted and APP mRNA level was measured by semi-quantitative PCR with specific primers. β-actin served as an internal control. 24-hourand 48-hourtreatment of high glucose did not significantly affect APP mRNA. Quantification of full-length APP after 24-hour treatment of high glucose (**D**)The values are expressed as mean±S.E.M, n = 7, by ANOVA. Quantification of full-length APP after 48-hour treatment of high glucose (**F**)The values are expressed as mean±S.E.M, n = 5, by ANOVA.

To examine whether the increase in full-length APP protein induced by high glucose treatment was via upregulation of *APP* gene transcription, we measured *APP* promoter activityin human neuroblastoma SH-SY5Y cells treated with 10 or 25 mM glucose. pAPP-Lucplasmid was constructed by inserting a 2.94 kb region of human *APP* promoter into the promoterless vector, pGL3-basic, upstream of the firefly luciferase reporter gene [32]. We found that neither 24-hour nor 48-hour treatment of 10 or 25 mM glucoseaffected the *APP* promoter activity (Fig. 2A and B).

Although the luciferase assay is very sensitive to assess *APP* promoter activity, the 2.94 kb promoter region of *APP* gene may not reflect the endogenous *APP* promoter activity. To further investigate if high glucose could affect APP transcription, weexamined the level of endogenous *APP* mRNA in SH-SY5Y cells after 10 or 25 mM glucose treatment. Consistent with the promoter assay data, 24-hour and 48-hour treatment with 10 or 25 mM glucose did not havesignificanteffect on *APP* mRNA level (Fig. 2C and E). Statistical analysis showed that there was no significant difference between glucose treatmentsand control (Fig. 2D and F).

### High Glucose Treatment Inhibits APP Degradation

**Figure 3:**
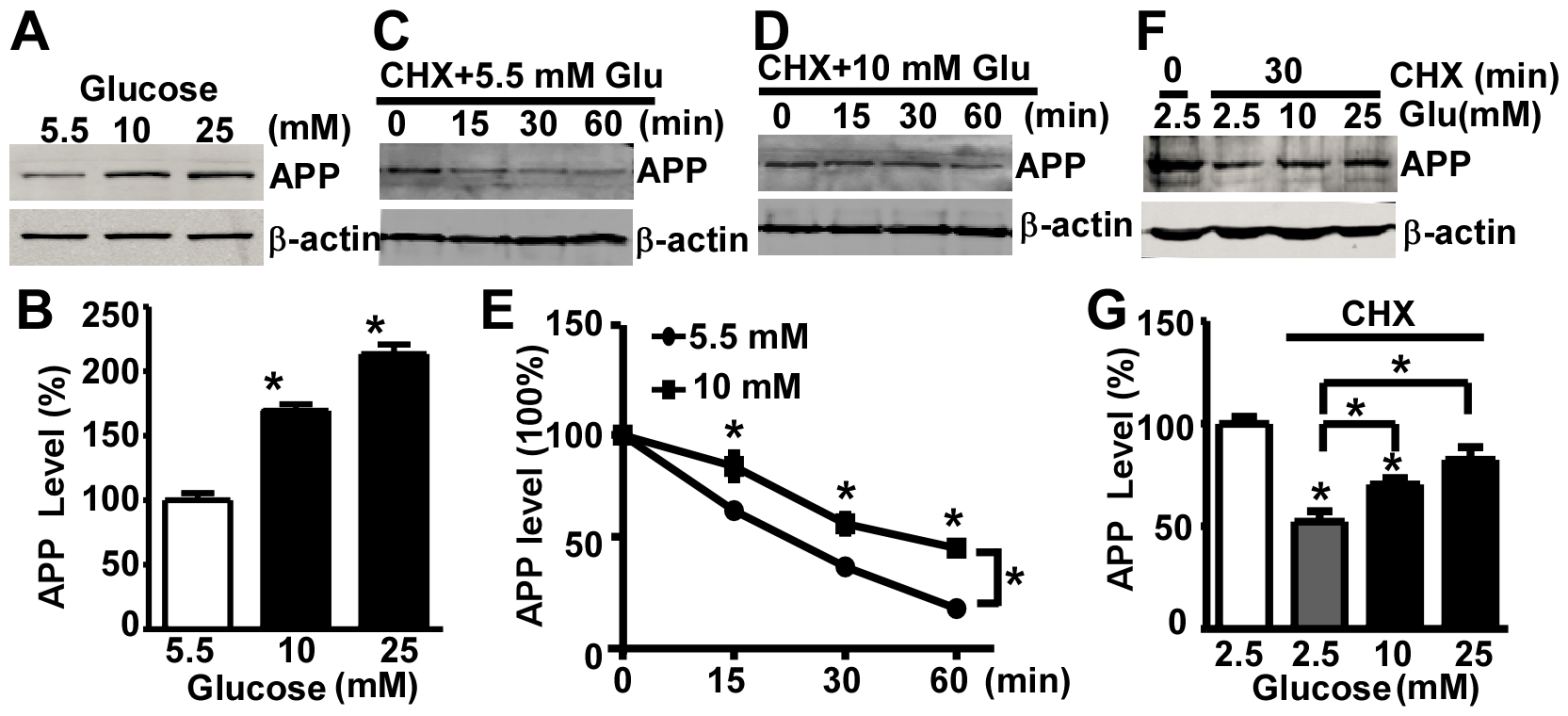
High glucose treatment inhibits APP protein degradation. 20E2 cells were treated with 5.5 mM, 10 mM or 25 mM glucose for 24 hours, and the cell lysates were analyzed by Western blot (**A**). Full-length APP was detected by C20 antibody. β-actin, serving as internal control, was detected by AC-15 antibody. The level of APP protein was quantified by Image J (**B**). The values are expressed as mean±S.E.M. n = 3,*p<0.0001, by ANOVA**.** For APP degradation experiment, 20E2 cells were treated with culturing media containing 100 ug/ml CHX along with 5.5 mM or 10 mM glucose. The cell lysates were harvested at 0, 15, 30 or 60 minutes after treatment and analyzed by Western blot (**C, D**).Quantification of APP protein by Image J (**E**). APP protein level was plotted as a percentage of the amount at 0 minute. The values are expressed as mean±S.E.M. n = 3,*p<0.001, by two-way ANOVA. The APP degradation experiment was also conducted using SH-SY5Y cells treated with 100 ug/ml CHX along with 5.5 mM, 10 mM or 25 mM glucose for 30 minutes. The cell lysates were analyzed by Western blot (**F**).The level of APP protein was quantified by Image J(**G**) andwas plotted as a percentage of the amount at 0 minute. The values are expressed as mean±S.E.M. n = 3,*p<0.05, by ANOVA.

Previous results have shown an increase in APP protein level yet without significant changein mRNA level. This dissociation between mRNA and protein level suggests that high glucose-induced alteration of APP level occurs at post-transcription level. To confirm it, we examined if high glucose treatment has effect on exogenous APP level in 20E2 cells, whose transcription was driven by the CMV promoter and therefore is not subject to transcriptionalregulation in human cells. 20E2 cell is a HEK cell line that stably expresses Swedish mutant APP_695_ under the CMV promoter. Since 20E2 derives from a peripheral cell line, we used 5.5 mM glucose, the physiological normal blood glucose at periphery, as control.Like in SH-SY5Y cells, we observed a significant increase in APP protein level in 20E2 cells after 24-hour high glucose treatment (Fig. 3A). The APP protein in cells treated with 10 mM glucose increased to 169.45±4.94% of that of the control group and to 213.36±7.33% in 25 mM group (F = 90.74, p<0.0001)(Fig. 3B). Theprotein level in the cells depends on the counterbalance between its production and degradation, and our data (Fig. 3A) indicated that increased APP translation or reduced APP degradation might be involved in high glucose-induced increase of APP expression. APP is degraded through proteasome and lysosome pathways [28]–[30], and both pathways have been reported to be altered by hyperglycemia [60], [61]. Therefore,we next examined ifhigh glucose-induced APP elevation could result from dysregulation of its degradation. We used cycloheximide (CHX) to stop protein synthesis and measured the amount of remaining protein at various time points after 10 or 25 mM glucose treatment. In 20E2 cells, elevated glucose concentration significantly enhanced APP level in a dosage dependent manner (F = 79.2, p<0.001). 15 minutes after CHX treatment, 65.57±1.67% of the initial total APP protein remained in cells treated with 5.5 mM glucose while 85.72±4.87% of initial APP persisted in 10 mM glucose treated cells (p<0.001) (Fig. 3C, D and E). At 30 minutes, cells cultured in 5.5 mM glucose media contained 39.94±0.21% of the initial total APP while cells treated with 10 mM glucose contained 59.57±3.28% of initial total APP (p<0.001) (Fig. 3C, D and E). At 60 minutes time point, the levels of APP were reduced to 20.91±0.08% and 48.38±0.88% of controls in cells treated with 5.5 mM and 10 mM glucose, respectively (p<0.001) (Fig. 3C, D and E).Similarly, in SH-SY5Y cells, 30 minutes after CHX treatment, 52.25±5.07% of the initial total APP protein remains in cells treated with 2.5 mM glucose while 70.48±2.95% APP persists in 10 mM glucosetreated cells, and 82.43±6.19% APP persists in 25 mM glucose treated cells (F = 6.58, p<0.05) (Fig. 3F and G). Taken together, our data suggested that high glucose slowed down the APPprotein degradation in both neuronal-like cells and non-neuronal cells.

### High Glucose Treatment Enhances C99 and Aβ Production

**Figure 4:**
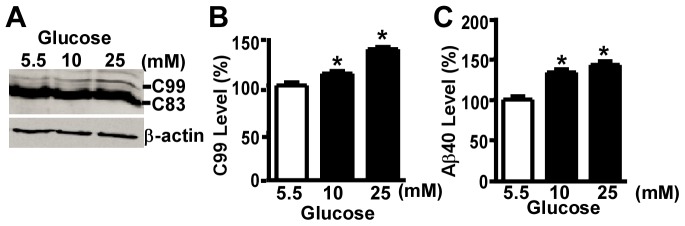
High glucose treatment increases C99 and Aβ_40_ production. The 20E2 cells were cultured and treated with different concentrations of glucose for 24 hours. Media containing 5.5 mM glucose served as control. The cell lysates were analyzed by western blot (**A**).C99 was detected by C20 antibody. β-actin, serving as internal control, was detected by AC-15 antibody. Quantification of C99 after 24-hour treatment of high glucose in 20E2 cells (**B**) The values are expressed as mean±S.E.M, n = 3,*p<0.001 by ANOVA The level of Aβ_40_ in conditioned media of 20E2 cells was measured by ELISA(**C**). The values are expressed as mean±S.E.M, n = 4, *p<0.001, by ANOVA.

APP is cleaved by BACE1 to generate C99 fragmentsand C99 is subsequently processed by γ-secretase to produce Aβ.Since the level of full-length APP was increased after high glucose treatment, we further examined whether high glucose treatment also has an impact on APP processing and Aβ production. We measured the level of C99 fragment and Aβ_40_ in the 20E2 cells. After 24-hour treatment, the level of C99 fragment significantly increased to 112.61±2.57% in cells treated with 10 mM glucose and to 138.33±2.18% in cells treated with 25 mM glucose (F = 52.46, p<0.001) (Fig. 4A and B).Moreover, the Aβ_40_ level increased to 133.21±3.69%at 10 mM glucose compared with control and to 142.49±4.21% when treated with culture media containing 25 mM glucose (F = 31.66, p<0.001) (Fig. 4C).Thus, high glucose treatment markedly enhanced the level of C99 and Aβ.

## Discussion

Diabetes has been shown to nearly double the risk of AD development [50], [51]. Yet the molecular mechanism underlying this association is not known. As a cardinal pathology of diabetes, chronic hyperglycemia is found to be inversely correlated with cognitive function [62], [63]. Meanwhile, AD is associated with hyperglycemia [56], [64], [65], indicating that hyperglycemia may play a role in AD pathogenesis.

Previous report has shown that people with diabetes have higher APP level in platelets than controls [66]. However, the role of high glucose in regulating expression of APP in neuronal cells remains elusive. Therefore, we first examined the effect of high glucose on APP expression in human neuroblastoma cells. We found that the level of APP protein is significantly increased by high glucose treatment for 24 or 48 hours (Fig. 1). To avoid the potential confounding effect of osmotic pressure, the osmolarity of all the treatment media was adjusted with D-mannitol. In fact, we found that treatment with osmolarity-unadjusted media induced a similar increase in APP protein as that by osmolarity-adjusted media (data not shown). Our data indicated that increased APP protein level by high glucose treatment is independent of high glucose-induced alteration of the osmotic pressure.

It has been shown that high glucose leads to excessive production of reactive oxygen species in neurons [67] and increased oxidative stress could activate NF-κB, which is involved in regulation of APP transcription [15]. Thus, we examined whether high glucose affects the activity of *APP* promoter region containing NF-κB sites. We found that both 24-hour and 48-hour high glucose treatmentdid not affect *APP* promoter activity (Fig. 2). In agreement with our result, previous study reported that reactive oxygen species did not activate the *APP* promoter in neurons [68]. To further confirm the effect of high glucose on global *APP* transcription, we measured *APP* mRNA level in cells treated with high glucose media. Consistently, high glucose treatment had no significant effect on the level of *APP* mRNA (Fig. 2). Our work first clarified that high glucose has no effect on *APP* transcription, at least with short term treatment.

Our result indicates that the increase in APP level induced by high glucose treatment occurs at posttranscriptional level, caused by enhanced APP translation or reduced APP degradation. APP protein undergoes rapid turnover as more than 70% of newly synthesized APP is intracellularly degraded [69], [70]. It is conceivable that reduction in APP degradation could lead to a significant increase in APP level, which is highly supported by the following evidence. First, it has been reported that proteasome and lysosome pathway are altered by hyperglycemia [60], [61] and APP is degraded through both pathways [28]–[30]. In addition, high glucose might affect APP modification, including glycosylation, phosphorylation and ubiquitination, which could lead to alteration of APP degradation and Aβproduction. Our data confirmed that high glucose treatment prolonged APP half-life from 25 minutes (5.5 mM) to around 60 minutes (10 mM) in HEK293 cells. Same trend was found in human neuroblastoma cells. The mechanism whereby high glucose could inhibit APP degradation remains to be clarified. It is possible that glycosylation modification induced by high glucose treatment is one of the mediators. APP is a glycoprotein that undergoes N-glycosylation and O-glycosylation during its passes through the endoplasmic reticulum and Golgi apparatus [23]. Studies using mannosidase inhibitors [71], [72] and mutation of glycosylation sites [73] demonstrated a significant impact of APP glycosylation on its trafficking and processing. Interestingly, it has been reported that APP degradation pathway could be altered by its glycosylation state which may subsequently induce conformational changes [74]. Moreover, N- and O-glycosylation predispose APP protein to Thr668 phosphorylation which, in turn, directs APP to axonal transportation [75]. The intracellular trafficking of APP is known to be an important determinant for its processing [76], [77]. Therefore, high glucose may affect APP processing and Aβ production through glycosylation and phosphorylation of APP which lead to alterations in intracellular trafficking and/or the conformation of the protein. In our western blots of full length APP, we did observe an upper APP band which could be glycosylated form. It is possibly caused by the following reasons. First, it has been shown that the signal of modified APP including glycosylated APP is much weaker than unmodified APP. Thus, normally, modified APP may not be easy to be detected. Second, the glycosylated APP has similar migration rate as unmodified APP or differentially modified APP. Elevated level of APP may mask the slight difference of the migration between glycosylated APP and unmodified APP. Although the glycosylated APP has not been detected, it is still possible that this modification contributes to the reduction of APP degradation and the elevation of Aβ level. In addition, impairment of proteasome or lysosome by high glucose may also contribute to the decrease of APP turnover rate. Moreover, as C99 and Aβ are degraded through proteasome pathway, high glucose-induced proteasomal impairment may inhibit C99 and Aβdegradation, which further leads to elevated level of C99 and Aβ [78]–[81]. Furthermore, the degradation of Aβ may also be reduced by high glucose-induced inhibition of insulin-degrading enzyme (IDE), which not only catalyzes the catabolism of insulin but also has been demonstrated to degrade Aβ both *in vivo* and *in vitro* [82]–[85]. Ffurther study is needed to clarify the underlying mechanism.

Increased APP expression contributes to Aβ overgeneration. Consistently, our data showed that high glucose-induced APP upregulation was highly associated with Aβ overproduction. The increase in Aβ production could result from enhanced APP level, yet, it is possible that high glucose may also upregulate β-secretaseand γ-secretase expression and/or activity contributing to Aβ overproduction. Hyperglycemia is known to cause elevated oxidative stress [86] and both BACE1 and presenilin 1 expression are known to be stimulated by oxidative stress accompanied by an increase in Aβ production [87]–[90]. Therefore, it could be helpful to further investigate the effect of high glucose on BACE1 and presenilin1. Furthermore, *in vivo* studies by using experimental animals may provide deeper insight into the effect of hyperglycemia on AD pathogenesis and underlying mechanisms.

In conclusion, we found that high glucose increases APP level through inhibition of its degradation and facilitates Aβ production. Our study provides a potential molecular mechanism underlying the association between diabetes and AD with the implication that glycemic control might be potentially beneficial for reducing the incidence of AD development in patients with DM and delaying the AD progression. Future *in vivo* studies will be essential to determine the effect of hyperglycemia on AD pathogenesis and the beneficial effect of glycemic control on reducing the incidence of AD development or slowing down the progression of AD.
